# Periodontal Dysbiosis and Premature Atherosclerosis: A Critical Appraisal of the Oral–Gut–Vascular Axis

**DOI:** 10.3390/biomedicines14092051

**Published:** 2026-09-12

**Authors:** Larisa Anghel, Dan-Nicolae Bele, Radu Andy Sascău, Laura-Cătălina Benchea, Monica Mihaela Scutariu, Cristina Prisacariu, Rodica Radu, Mircea Ovanez Balasanian, Cristian Stătescu

**Affiliations:** 1 Internal Medicine Department, “Grigore T. Popa” University of Medicine and Pharmacy, 700503 Iași, Romaniaovanes718@yahoo.com (M.O.B.);; 2 Cardiology Department, Cardiovascular Diseases Institute “Prof. Dr. George I. M. Georgescu”, 700503 Iași, Romania; danbele2000@gmail.com; 3 Department of Oral Diagnosis, Faculty of Dental Medicine, “Grigore T. Popa” University of Medicine and Pharmacy, 700115 Iași, Romania

**Keywords:** premature coronary artery disease, periodontitis, oral–gut axis, gut barrier dysfunction, dysbiosis, endotoxemia, TMAO, endothelial dysfunction, atherosclerosis, precision microbiome medicine

## Abstract

**Background/Objectives**: Premature coronary artery disease (PCAD) is not fully explained by conventional risk factors, particularly in younger adults with residual inflammatory risk. This review evaluates the oral–gut–vascular axis as a mechanistic framework linking periodontal dysbiosis to premature atherosclerosis. **Methods**: A structured narrative search of PubMed/MEDLINE, Scopus, Embase, and Web of Science was conducted for English-language publications issued between January 2015 and June 2026. Human observational and interventional studies, mechanistic studies, systematic reviews, meta-analyses, and major scientific statements addressing periodontal disease, gut dysbiosis, barrier dysfunction, microbial metabolites, and vascular outcomes were considered. **Results**: Current evidence supports the biological plausibility of interconnected pathways involving periodontal pathobionts, microbial translocation, intestinal dysbiosis, increased epithelial permeability, endotoxemia, TLR2/TLR4 signaling, TMAO metabolism, oxidative stress, immune dysregulation, endothelial dysfunction, and plaque development. Candidate translational markers include hsCRP, IL-6, LPS, LBP, TMAO, oxidized LDL, adhesion molecules, salivary microbial signatures, and vascular imaging indices. Periodontal treatment and microbiome-directed strategies may reduce inflammatory burden, although effects on major cardiovascular outcomes remain unproven. **Conclusions**: The oral–gut–vascular axis is biologically plausible but not yet causally established in humans, and its specific relevance to PCAD remains largely inferential. Prospective PCAD-specific cohorts, standardized multi-omics, validated biomarkers, vascular imaging, and externally validated artificial-intelligence models are required to establish clinical utility and inform integrated cardio-dental prevention in younger populations.

## 1. Introduction

Premature coronary artery disease (PCAD) is usually defined as obstructive or clinically relevant coronary artery disease occurring before 55 years of age in women and before 45 years of age in men, although the thresholds used in epidemiological studies are not uniform [1,2]. Despite this variation, PCAD represents a clinically important phenotype because vascular disease develops before the prolonged lifetime exposure to cardiometabolic risk factors typically observed in older patients. Ischemic heart disease is already a major contributor to mortality in young and middle-aged adults, and the Global Burden of Disease 2019 analysis identified ischemic heart disease as one of the leading causes of death among individuals aged 25–49 years [3].

Classical risk factors, including smoking, obesity, dyslipidemia, arterial hypertension, diabetes mellitus and family history, remain central to PCAD pathogenesis [1,2]. Nevertheless, these factors do not fully account for the occurrence, severity or recurrence of early coronary events. The persistence of major adverse cardiovascular events despite lipid-lowering therapy, antihypertensive treatment, smoking cessation and lifestyle optimization has led to the concept of residual cardiovascular risk [4]. Residual risk includes thrombotic, lipid-related, metabolic and inflammatory components; among these, chronic low-grade inflammation has become particularly relevant for younger individuals in whom long cumulative exposure to conventional risk factors may be limited.

The human microbiome has emerged as a systemic regulator of immune, metabolic and vascular homeostasis. Gut microbial communities influence host physiology through microbial metabolites, bile acid transformation, epithelial barrier regulation, mucosal immune signaling, systemic inflammatory tone and interactions with hepatic metabolism. In the setting of residual inflammatory risk, microbiome dysbiosis may provide a biologically plausible, modifiable and clinically underrecognized contributor to premature atherogenesis [5].

The oral cavity represents a major microbial reservoir with direct access to the systemic circulation and indirect access to the intestinal ecosystem. Periodontal pockets provide an anaerobic inflammatory niche that favors expansion of pathobionts and repeated release of microbial products. Oral pathogens and their virulence factors may disseminate through hematogenous routes, inflammatory spillover and swallowing-mediated enteric translocation. These routes connect periodontal inflammation with gut microbial remodeling, intestinal barrier disruption, endotoxemia and vascular injury [6,7].

Longitudinal evidence supports the long-term vascular relevance of oral infections. In a cohort followed from childhood into adulthood, oral infections identified early in life were associated with subclinical carotid atherosclerosis decades later, even after adjustment for cumulative cardiovascular risk exposure [5]. This observation is important because it suggests that oral inflammatory burden may contribute to vascular aging before overt cardiovascular disease becomes clinically apparent.

This review proposes the oral–gut–vascular axis as an integrated mechanistic framework explaining how periodontal dysbiosis may contribute to premature atherosclerosis. Unlike reviews focused exclusively on periodontitis or on gut microbiota, this manuscript emphasizes the continuum from oral dysbiosis to gut barrier dysfunction, microbial metabolite remodeling, systemic inflammation, endothelial injury and early coronary disease. The review is aligned with the thematic scope of precision microbiome modulation by focusing on barrier function, metabolic inflammation, biomarkers and clinically translatable interventions.

Importantly, the evidentiary layers supporting this framework are not equivalent. Observational data support an association between periodontitis and atherosclerotic cardiovascular disease, whereas evidence for gut-mediated causation and for PCAD-specific clinical relevance is substantially less direct. This review therefore evaluates convergence, directness, and translational limitations rather than treating biological plausibility as proof of causality.

The oral–gut–vascular axis should also be interpreted within the broader contemporary framework of periodontal medicine. Romandini et al. recently proposed seven non-mutually exclusive mechanisms through which periodontitis may influence systemic disease, including microbial translocation, systemic meta-inflammation, maladaptive myelopoiesis, trafficking of immune players, masticatory-dysfunction-mediated dietary alterations, functional dysregulation of the oral microbiome, and shared underlying vulnerabilities. Within this broader framework, the present review focuses specifically on pathways most directly relevant to vascular injury: microbial translocation, oral–gut ecological disruption, systemic inflammatory amplification, microbial metabolite remodeling, immune and oxidative dysregulation, and endothelial injury. Thus, the oral–gut–vascular axis should be viewed as a disease-specific integrative model rather than as an alternative to the broader periodontal medicine framework [8].

**Figure 1:**
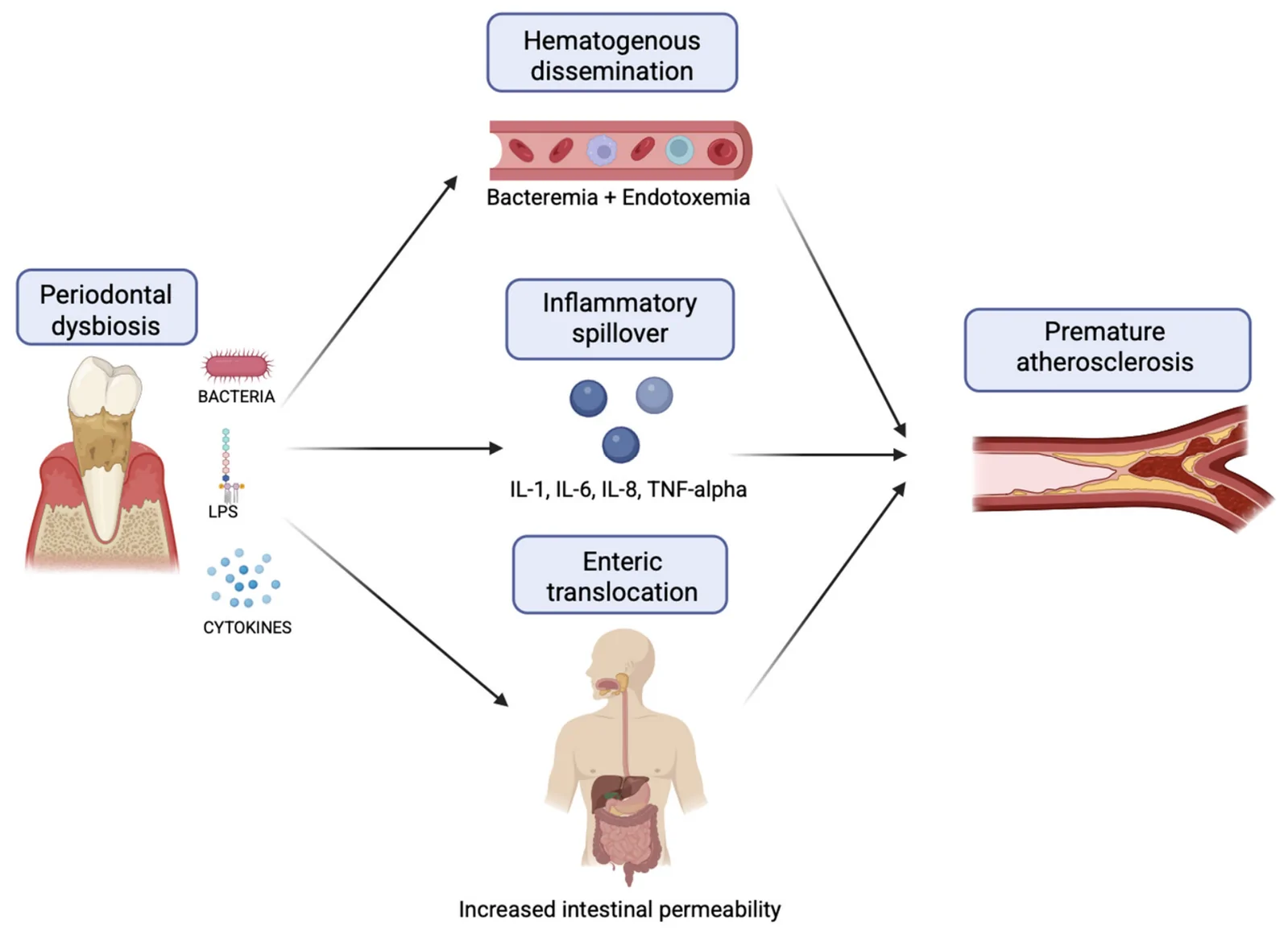
The oral–gut–vascular axis in premature atherosclerosis. IL-1, interleukin-1; IL-6, interleukin-6; IL-8, interleukin-8; LPS, lipopolysaccharide; TNF-α, tumor necrosis factor alpha.

The working hypothesis is that periodontal dysbiosis acts as an upstream inflammatory and microbial trigger that can reshape intestinal ecology and host–microbe interactions. Through increased intestinal permeability, circulating lipopolysaccharide (LPS), trimethylamine N-oxide (TMAO), Toll-like receptor (TLR) activation, oxidative stress and endothelial dysfunction, this axis may amplify premature atherosclerosis, particularly in young adults with unexplained or disproportionate residual inflammatory risk (Figure 1).

The objective was not to estimate a pooled effect, but to critically examine the convergence, directness, and translational limitations of the evidence supporting the proposed oral–gut–vascular axis in PCAD.

## 2. Literature Search Strategy

A structured narrative literature search was conducted in PubMed/MEDLINE, Scopus, Embase, and the Web of Science Core Collection to identify relevant publications issued between 1 January 2015 and 30 June 2026. The final search was updated on 30 June 2026. The search strategy combined controlled vocabulary, where available, and free-text terms related to (“periodontitis” OR “periodontal dysbiosis” OR “oral microbiome”) AND (“gut microbiome” OR “oralization” OR “intestinal permeability” OR “barrier dysfunction”) AND (“atherosclerosis” OR “coronary artery disease” OR “premature coronary artery disease”). Additional search terms included “*Porphyromonas gingivalis*”, “microbial translocation”, “lipopolysaccharide”, “endotoxemia”, “TLR2”, “TLR4”, “TMAO”, “endothelial dysfunction”, “vascular imaging”, “microbiome modulation”, and “periodontal treatment”.

Sources considered included human observational and interventional studies, mechanistic animal studies, systematic reviews, meta-analyses, and major scientific statements published in English. Priority was given to longitudinal human studies, controlled interventional studies, multi-omics investigations, and studies reporting validated endothelial, vascular, or imaging endpoints. Reference lists of eligible publications were manually screened to identify additional relevant studies. Seminal publications predating 2015 were considered for historical or mechanistic context but were not part of the core 2015–June 2026 evidence base.

Owing to substantial heterogeneity in study populations, periodontal definitions, microbiome sequencing and analytical methodologies, biomarker measurements, and vascular outcomes, the evidence was synthesized narratively rather than quantitatively.

Because this is a structured narrative review, no formal meta-analysis, risk-of-bias assessment, or GRADE certainty rating was undertaken. Evidence was appraised qualitatively according to study design, directness, consistency, biological coherence, and relevance to PCAD. Human longitudinal and interventional data were weighted more heavily than cross-sectional observations, while animal and in vitro findings were interpreted as mechanistic support rather than clinical confirmation. The synthesis explicitly distinguishes three evidence layers: the general periodontitis–atherosclerotic cardiovascular disease (ASCVD) association, evidence for oral-to-gut mediation, and evidence specific to premature coronary disease.

The search was designed to identify representative and clinically relevant evidence rather than to provide an exhaustive systematic synthesis. Study selection was iterative and guided by methodological quality, relevance to the proposed mechanistic framework, and directness to premature coronary artery disease.

Given the structured narrative design of this review, study selection was not conducted according to a formal systematic-review screening framework; instead, evidence was prioritized according to methodological quality, mechanistic relevance, directness, and relevance to premature coronary artery disease.

## 3. Periodontal Dysbiosis as the Initial Trigger of Systemic Inflammation

### 3.1. Epidemiology and Pathobiology of Periodontitis

Periodontitis is a chronic, infection-driven inflammatory disease of the tooth-supporting tissues, including gingiva, periodontal ligament, cementum and alveolar bone. It develops through a complex interaction between the oral microbiome and the host immune response, with genetic, behavioral, environmental and metabolic factors modifying susceptibility and progression [9,10]. Clinically, the disease is defined by irreversible periodontal attachment loss and alveolar bone destruction, ultimately leading to tooth mobility and tooth loss when un-treated [9].

The global burden of periodontal disease is substantial. Recent epidemiological data indicate that periodontal diseases affect approximately 19% of adults worldwide and account for more than one billion cases, while severe periodontitis remains among the most common chronic non-communicable diseases [10]. Global Burden of Disease 2021 estimates suggest more than 790 million prevalent cases of periodontal disease, demonstrating its persistent population-level impact despite improvements in several oral health indicators [11].

The modern understanding of periodontitis has moved away from a linear, single-pathogen infection model. In periodontal health, the oral microbiome exists in dynamic equilibrium with the host, and commensal organisms contribute to local immune tolerance and tissue homeostasis. In susceptible individuals, ecological disruption of the subgingival biofilm promotes expansion of pathobionts, increased microbial diversity within anaerobic niches, and a maladaptive inflammatory response. Deepening periodontal pockets further reinforce an anaerobic environment that sustains pathogenic communities and accelerates tissue destruction [9,10,12].

### 3.2. Periodontal Pathobionts and Virulence Factors

Among periodontal pathobionts, *Porphyromonas gingivalis* has received particular attention because of its ability to remodel microbial communities and subvert host immunity. Rather than acting only as a conventional pathogen, *P. gingivalis* behaves as a keystone organism capable of altering the inflammatory and ecological balance of the periodontal niche. Other organisms, including *Tannerella forsythia*, *Treponema denticola* and *Prevotella intermedia*, may act synergistically within dysbiotic communities and contribute to persistent inflammation and tissue injury [13].

*P. gingivalis* expresses multiple virulence determinants relevant to systemic disease. Fimbriae promote adhesion, epithelial invasion and biofilm maturation; LPS modulates innate immune signaling; gingipains degrade structural and immune proteins; and outer membrane vesicles (OMVs) disseminate proteolytic enzymes, LPS and other bioactive molecules beyond the local biofilm [13]. These factors facilitate immune evasion, bacterial persistence, vascular exposure and systemic inflammatory activation. In addition, periodontal procedures, mastication and routine oral hygiene in the presence of inflamed periodontal tissues can promote transient bacteremia and release of microbial products into the circulation.

Periodontal virulence factors may influence vascular biology directly and indirectly. LPS can activate TLR-dependent signaling, gingipains may enhance tissue breakdown and inflammatory amplification, and OMVs may serve as nanoscale vehicles delivering bacterial components to distant tissues. These processes can stimulate cytokine release, oxidative stress, endothelial activation and prothrombotic responses. Accordingly, periodontal dysbiosis should be considered not simply as a local oral condition, but as a chronic source of microbial and inflammatory exposure with systemic consequences [13].

Importantly, LPS bioactivity is not uniform across periodontal Gram-negative organisms. Structural variation in the lipid A moiety influences recognition by the TLR4/Myeloid Differentiation Protein 2 (MD-2) complex and can substantially modify the magnitude and pattern of downstream NF-κB and MAPK signaling. Accordingly, the inflammatory consequences of periodontal endotoxemia depend not only on circulating LPS concentration but also on its microbial origin and structural configuration. In addition to *P. gingivalis*, *Fusobacterium nucleatum* represents an important periodontal pathobiont with systemic relevance. Its LPS can elicit potent innate immune activation, while its FadA adhesin enables binding to vascular endothelial cadherin, increases endothelial permeability, and facilitates bacterial translocation across endothelial barriers [14].

### 3.3. Systemic Inflammatory Spillover

A defining feature of periodontitis is its ability to generate a persistent low-grade inflammatory state. Patients with periodontitis frequently exhibit increased circulating concentrations of C-reactive protein (CRP), tumor necrosis factor-alpha (TNF-α), interleukin (IL)-1, IL-6, IL-8 and leptin, supporting the view that periodontal disease is not restricted to the oral cavity [6]. These mediators are also relevant to atherosclerosis, where they promote leukocyte recruitment, endothelial activation, plaque inflammation and thrombosis.

Systemic spillover may occur through several, partially overlapping mechanisms: recurrent bacteremia, dissemination of microbial products, release of inflammatory cytokines from periodontal tissues, activation of neutrophils and monocytes, and changes in host lipid and oxidative pathways. The chronicity of periodontal inflammation is particularly important. Unlike an acute infection, periodontitis may expose the host to repeated microbial and inflammatory stimuli over years, thereby contributing to cumulative vascular injury and residual inflammatory risk.

### 3.4. Homeostatic Functions of the Oral Microbiome: The Nitrate–Nitrite–NO Pathway

The oral microbiome also performs homeostatic functions that may support vascular physiology. A prominent example is the enterosalivary nitrate–nitrite–nitric oxide pathway. Dietary nitrate is concentrated in saliva and reduced to nitrite by nitrate-reducing oral bacteria; nitrite can subsequently contribute to systemic nitric oxide generation, particularly under conditions in which oxygen-dependent nitric oxide synthase activity is limited. This pathway has been linked to vascular tone, endothelial function, and blood-pressure regulation. Periodontal dysbiosis or indiscriminate suppression of the oral microbiota may theoretically impair this metabolic function. Antiseptic mouthwashes, particularly chlorhexidine-containing formulations, have been reported to reduce oral nitrate-reducing activity and salivary nitrite concentrations, with some studies also reporting modest increases in blood pressure. However, findings are heterogeneous, intervention periods are generally short, and evidence relating these changes to hard cardiovascular outcomes is lacking. Accordingly, preservation or restoration of beneficial microbial functions represents an emerging concept rather than an established cardiovascular therapeutic strategy [15].

## 4. The Oral–Gut Axis: Microbial Translocation, Colonization and Barrier Dysfunction

### 4.1. Oralization of the Gut Microbiome

The oral and gut microbiomes are anatomically connected through constant swallowing of saliva, oral bacteria and microbial products. Under physiological conditions, gastric acidity, mucosal immunity, bile acids, resident gut microbial communities and epithelial barrier mechanisms limit colonization by oral organisms. However, periodontal disease increases the oral microbial load and favors repeated delivery of pathobionts to the gastrointestinal tract [7].

The term oralization of the gut microbiome describes the enrichment of intestinal microbial communities with oral taxa. This phenomenon is increasingly recognized in systemic diseases characterized by chronic inflammation, metabolic disruption or impaired barrier integrity. In periodontitis, oralization may occur when periodontal pathogens survive gastric passage, interact with resident gut microorganisms and colonize intestinal niches. *P. gingivalis* and other oral taxa may contribute to gut dysbiosis by altering microbial diversity, reducing colonization resistance and promoting pro-inflammatory microbial functions [7].

Host-derived mediators may also participate in oral–gut ecological communication. Recent experimental studies implicate hepatocyte growth factor (HGF) as one such modifier. HGF overexpression has been shown to alter the periodontal microbial community in a disease-stage-dependent manner and, in experimental periodontitis, to aggravate intestinal microbial disruption and barrier dysfunction. In HGF-overexpressing mice with periodontitis, increased gut permeability was accompanied by higher circulating D-lactate and LPS concentrations and alterations in tight-junction-related markers. These findings suggest that host inflammatory mediators may influence both the microbial and epithelial components of the oral–gut axis. However, these data are experimental and should not yet be extrapolated to human cardiovascular risk [16,17].

### 4.2. Microbial Translocation and Intestinal Colonization

The enteric route of translocation begins with swallowing of periodontal bacteria and virulence factors. Although the stomach represents an important antimicrobial barrier, certain organisms may tolerate acidic conditions or survive within protective biofilm structures. Once in the intestine, oral-derived microbes can interact with resident communities, epithelial cells and mucosal immune compartments. These interactions may favor dysbiosis, particularly when environmental modifiers such as smoking, high-fat diet, antibiotic exposure or metabolic disease reduce microbial resilience [7,18].

Experimental data support this concept. In animal models, oral infection with *P. gingivalis* has been associated not only with aggravation of atherosclerosis but also with changes in gut microbial diversity, indicating bidirectional interaction between oral and intestinal microbial ecosystems [19]. Although translation to humans requires caution, these models provide mechanistic plausibility for an oral–gut pathway connecting periodontal inflammation to systemic vascular disease.

### 4.3. Gut Barrier Dysfunction and Metabolic Endotoxemia

The intestinal barrier is maintained by epithelial cells, mucus, antimicrobial peptides, immune surveillance and tight-junction proteins such as zonula occludens-1, occludin and claudins. Dysbiosis can impair these protective mechanisms, increase epithelial permeability and permit translocation of microbial-associated molecular patterns into the circulation. In this context, LPS derived from Gram-negative bacteria becomes a key mediator of metabolic endotoxemia and systemic immune activation [20,21].

Periodontal-induced gut dysbiosis may therefore amplify cardiovascular risk by weakening mucosal barrier integrity. In experimental periodontitis, intestinal structural abnormalities and increased circulating LPS concentrations have been reported, suggesting that periodontal inflammation may influence the gut barrier remotely [20]. LPS can then interact with immune cells, hepatocytes and vascular cells, thereby linking intestinal permeability with systemic inflammation, altered hepatic metabolism and endothelial dysfunction.

### 4.4. Environmental and Host Modifiers

The oral–gut axis is strongly influenced by environmental and host factors. The human microbiome begins to develop at birth and is shaped by maternal microbial exposure, infant feeding, immune maturation, diet, antibiotics, sleep, physical activity, stress, occupation, pet exposure and other lifestyle factors [18]. Although host genetics contribute to microbial variation, environmental exposures appear to be dominant determinants of microbial composition and function.

Diet is particularly relevant to the oral–gut–vascular axis. Dietary substrates such as choline, phosphatidylcholine and carnitine influence microbial production of trimethylamine (TMA), the precursor of TMAO. Conversely, fiber-rich dietary patterns may support short-chain fatty acid production, barrier integrity and anti-inflammatory immune regulation. Antibiotic exposure can produce persistent shifts in microbial ecology, while smoking and poor oral hygiene may intensify periodontal dysbiosis. These modifiers are clinically important because they represent potentially actionable targets for precision microbiome interventions [18].

### 4.5. Cardiometabolic Modifiers and Feed-Forward Amplification

The oral–gut–vascular axis is unlikely to operate as a unidirectional sequence. Periodontal inflammation, intestinal dysbiosis, systemic metabolic inflammation, and vascular dysfunction may reinforce one another through feed-forward interactions. Obesity, diabetes, dyslipidemia, smoking, dietary patterns, and other cardiometabolic exposures may increase both periodontal susceptibility and intestinal barrier dysfunction while independently promoting endotoxemia, oxidative stress, endothelial injury, and atherosclerosis. Conversely, chronic periodontal inflammation may further increase systemic inflammatory tone and worsen metabolic homeostasis [21].

**Figure 2:**
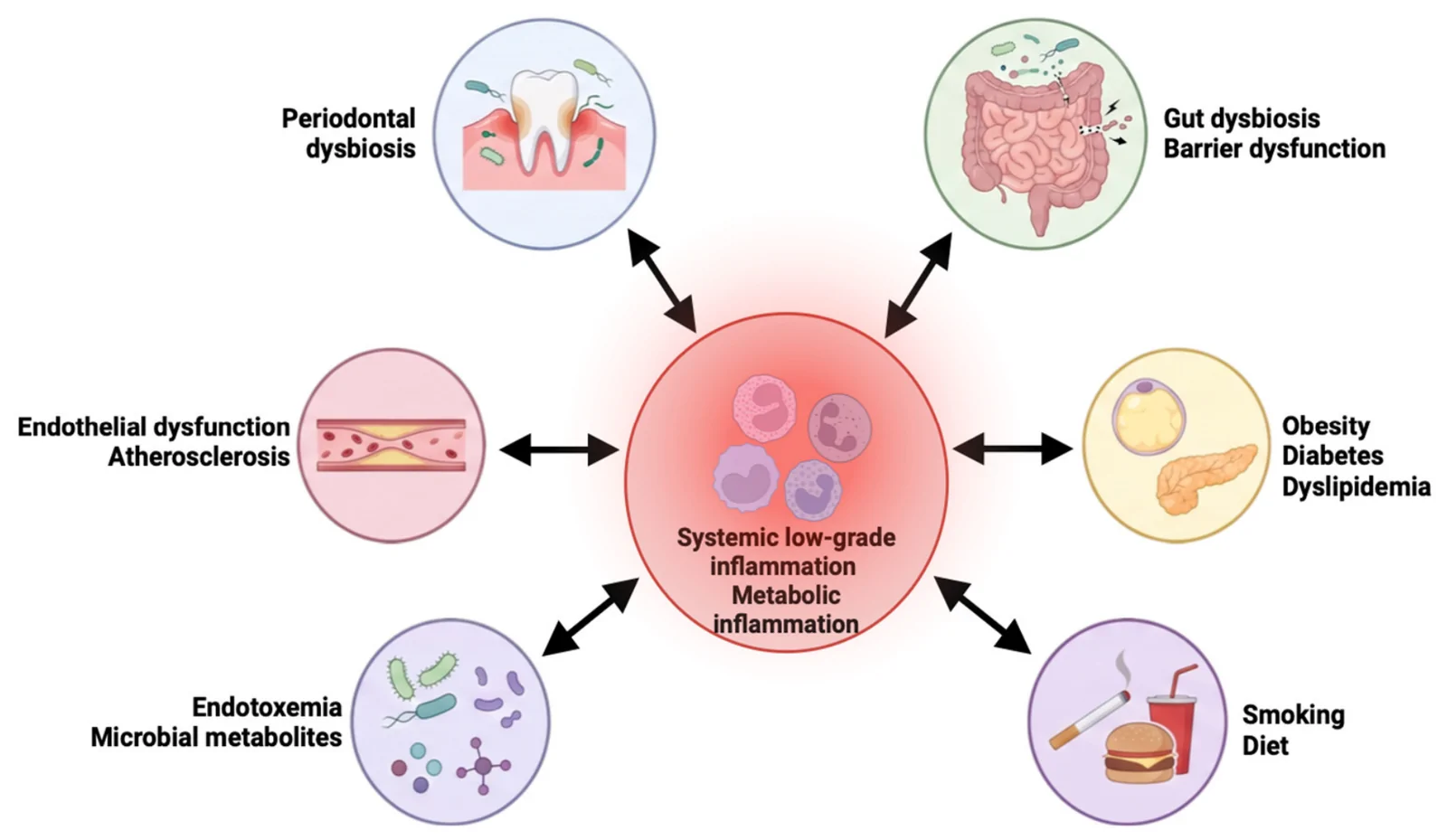
Bidirectional amplification model of the oral–gut–vascular axis.

These interactions are particularly relevant when interpreting experimental and observational data. The vascular impact of periodontal inflammation may differ substantially according to the underlying metabolic phenotype, and findings obtained in hyperlipidemic or hyperglycemic experimental models cannot necessarily be extrapolated to metabolically healthy hosts. Therefore, obesity, diabetes, dyslipidemia, and related exposures should be considered both potential confounders and biological effect modifiers in future mechanistic and clinical studies [20]. The oral–gut–vascular axis is more appropriately conceptualized as a dynamic bidirectional network than as a unidirectional pathway. In this model, periodontal dysbiosis, gut dysbiosis with barrier dysfunction, endotoxemia, microbial metabolites, and systemic low-grade/metabolic inflammation interact continuously and may reinforce one another over time. Cardiometabolic conditions such as obesity, diabetes, and dyslipidemia, as well as smoking and dietary factors, may intensify these interactions and increase susceptibility to endothelial dysfunction and atherosclerosis. Conversely, vascular and metabolic disease states may perpetuate inflammatory and microbial imbalance, creating a feed-forward loop. Figure 2 illustrates this bidirectional amplification model of the oral–gut–vascular axis.

## 5. Microbiome-Driven Mechanisms Linking Periodontal Dysbiosis to Vascular Injury

**Figure 3:**
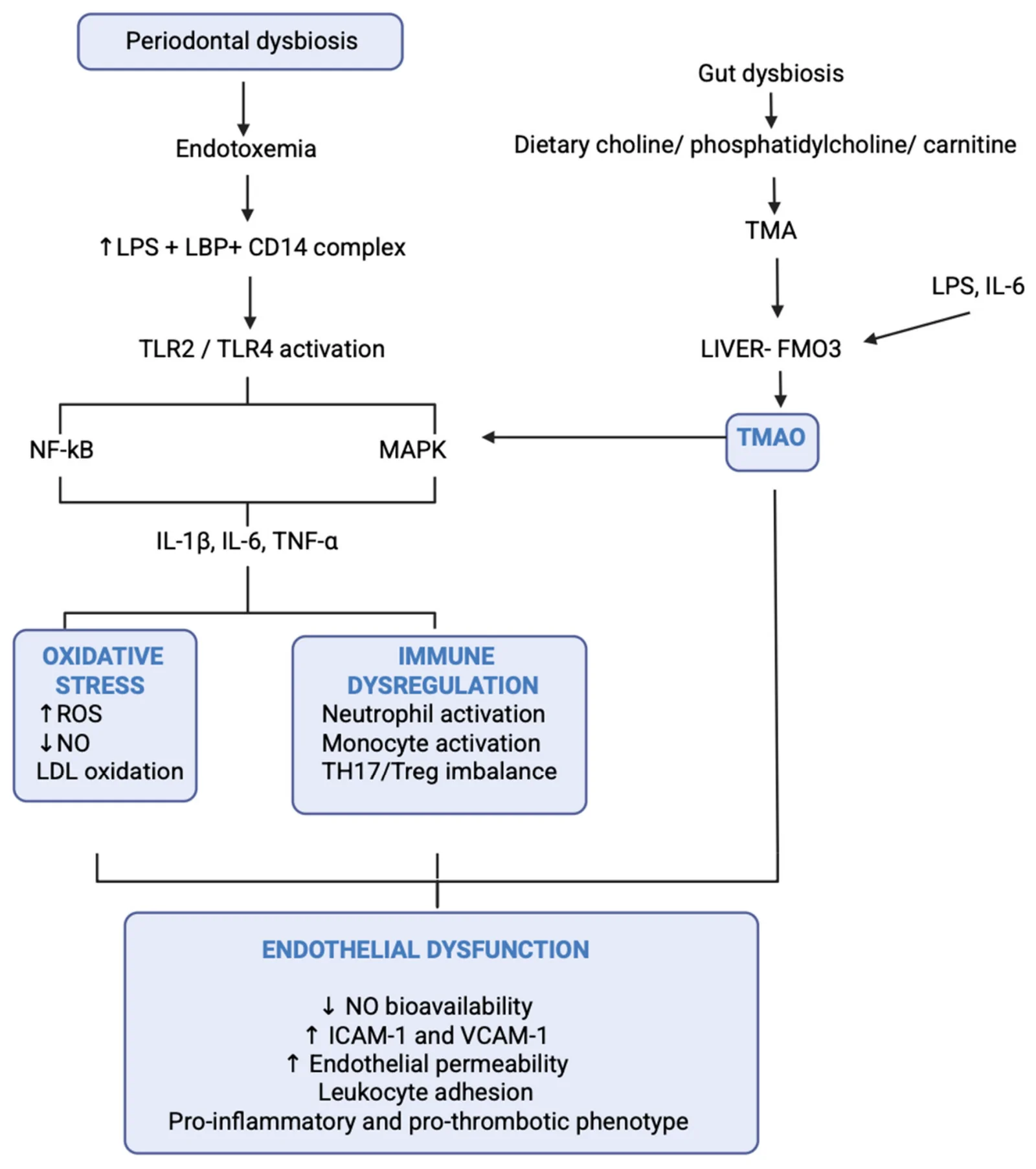
Mechanistic pathways linking periodontal dysbiosis to vascular injury. CD14, cluster of differentiation 14; FMO3, flavin-containing monooxygenase 3; ICAM-1, intercellular adhesion molecule 1; IL-1β, interleukin-1 beta; IL-6, interleukin-6; LBP, lipopolysaccharide-binding protein; LDL, low-density lipoprotein; LPS, lipopolysaccharide; MAPK, mitogen-activated protein kinase; NF-κB, nuclear factor kappa B; NO, nitric oxide; ROS, reactive oxygen species; Th17, T helper 17 cell; TLR2, Toll-like receptor 2; TLR4, Toll-like receptor 4; TMA, trimethylamine; TMAO, trimethylamine N-oxide; TNF-α, tumor necrosis factor alpha; Treg, regulatory T cell; VCAM-1, vascular cell adhesion molecule 1.

For conceptual clarity, the mechanisms discussed below are organized according to the pathway illustrated in Figure 3. Two major upstream microbial signals are considered: microbial products, particularly LPS, which engage LBP/CD14 and TLR2/TLR4-dependent inflammatory signaling, and microbiome-derived metabolites, particularly TMAO. These pathways converge through oxidative stress and immune dysregulation on endothelial dysfunction, which represents the principal vascular phenotype linking microbial dysbiosis to early atherogenesis. These pathways are interconnected rather than mutually exclusive.

### 5.1. Microbial Products, Endotoxemia and TLR2/TLR4 Signaling

Endotoxemia is a central mechanism connecting periodontal dysbiosis, gut barrier dysfunction and vascular inflammation. LPS derived from periodontal or intestinal Gram-negative bacteria can enter the circulation during bacteremia or after increased intestinal permeability. Circulating LPS binds to LPS-binding protein (LBP), interacts with CD14 and activates TLR4-dependent signaling in immune and vascular cells. This pathway promotes nuclear factor kappa β (NF-kβ) and Mitogen-Activated Protein Kinase (MAPK) activation, leading to production of IL-1β, IL-6, TNF-α and other pro-inflammatory mediators [21,22].

Beyond its immunologic effects, LPS may contribute to altered hepatic metabolism, endothelial activation and platelet reactivity. Chronic low-grade endotoxemia can maintain monocyte activation and increase the responsiveness of innate immune cells to subsequent stimuli. This trained or primed inflammatory phenotype is relevant to atherosclerosis because activated monocytes and macrophages participate directly in plaque formation, lipid uptake and cytokine production.

TLR2 and TLR4 are major pattern-recognition receptors involved in the host response to periodontal pathogens and their products [22]. TLR4 recognizes LPS and activates intracellular adaptor pathways that converge on NF-kβ and MAPK signaling. TLR2 also contributes to recognition of bacterial lipoproteins and other microbial components. In periodontal infection models, increased TLR2 and TLR4 expression has been observed, and genetic deficiency of these receptors attenuates infection-associated atherosclerotic plaque development [22].

These data support the concept that TLR signaling acts as a molecular bridge between microbial dysbiosis and vascular inflammation. Persistent activation of TLR pathways can increase endothelial adhesion molecule expression, stimulate cytokine production, recruit leukocytes into the vascular wall and promote macrophage foam-cell formation. In young individuals with PCAD, such mechanisms may be particularly relevant when atherosclerosis develops despite limited duration of exposure to conventional risk factors.

### 5.2. Microbial Metabolite Remodeling: TMA/TMAO

TMAO is one of the most extensively studied microbiome-derived metabolites linking gut dysbiosis with atherosclerosis. Intestinal microorganisms metabolize dietary choline, phosphatidylcholine and carnitine into TMA, which is subsequently oxidized in the liver by flavin-containing monooxygenases (FMO), particularly FMO3, to generate TMAO [20]. Experimental periodontitis induced by *P. gingivalis* has been shown to increase circulating TMAO concentrations while aggravating atherosclerotic plaque formation, suggesting that periodontal inflammation can affect cardiovascular risk through microbial metabolic remodeling [20].

TMAO may promote atherosclerosis through several mechanisms, including impaired cholesterol elimination, enhanced macrophage scavenger receptor expression, foam-cell formation, vascular inflammation, endothelial activation and platelet hyperreactivity [20,23]. TMAO also interacts with inflammatory signaling pathways, including MAPK and NF-kβ-related cascades. Importantly, oral *P. gingivalis* administration in experimental models altered gut microbial composition and increased hepatic FMO3 expression, indicating that the effect of periodontal disease extends beyond microbial composition to host hepatic metabolic pathways [20].

Inflammation may further intensify this metabolic loop. Increased hepatic IL-6 expression has been observed together with elevated FMO3 levels, and both LPS and IL-6 can upregulate FMO3 expression [20]. These findings support a feed-forward mechanism in which periodontal dysbiosis induces gut dysbiosis and endotoxemia, endotoxemia increases inflammatory signaling, inflammation enhances hepatic TMAO production, and TMAO further promotes vascular injury.

### 5.3. Oxidative Stress and Nitric Oxide Impairment

Oxidative stress is a convergent mechanism in periodontal disease, gut dysbiosis and atherosclerosis. Reactive oxygen species (ROS) generated during chronic inflammation can reduce nitric oxide (NO) bioavailability, uncouple endothelial nitric oxide synthase (eNOS), oxidize low-density lipoprotein (LDL) particles and amplify endothelial activation [24,25]. In the vasculature, reduced NO bioavailability impairs vasodilation and shifts the endothelium toward a pro-inflammatory, pro-thrombotic and proliferative phenotype.

Periodontitis may contribute to oxidative stress through persistent neutrophil activation, cytokine release, microbial LPS exposure and systemic inflammatory priming. The vascular consequences include increased endothelial permeability, leukocyte adhesion, platelet activation and smooth muscle cell migration. These processes are critical in the transition from early endothelial dysfunction to atherosclerotic plaque formation and progression [24,25,26] (Figure 3).

### 5.4. Immune Dysregulation and Inflammatory Amplification

Immune dysregulation provides another mechanistic link between periodontitis and atherosclerosis. Periodontal disease is associated with neutrophil activation, excessive release of proteolytic enzymes, oxidative burst, extracellular trap formation and chronic cytokine production [27]. These processes can spill over systemically and increase vascular inflammatory tone.

Macrophages are central to plaque development. Periodontal pathogens and their products can influence macrophage polarization, lipid uptake and cholesterol efflux. *P. gingivalis* has been implicated in foam-cell formation by altering macrophage lipid metabolism toward increased lipid accumulation and reduced cholesterol export [28]. T-cell imbalance may also contribute; altered Th17/Treg balance and indoleamine 2,3-dioxygenase activity have been described in periodontitis-associated atherosclerotic patients, suggesting adaptive immune involvement in the oral–vascular connection [29].

### 5.5. Endothelial Dysfunction as the Convergent Vascular Phenotype

Endothelial dysfunction represents an early, measurable and prognostically relevant event in atherogenesis. Under physiological conditions, the endothelium regulates vascular tone, leukocyte trafficking, thrombosis, smooth muscle cell function and inflammatory responses. Dysfunction is characterized by reduced NO bioavailability, imbalance between vasodilatory and vasoconstrictive mediators, increased oxidative stress, endothelial permeability and expression of adhesion molecules such as intercellular adhesion molecule 1 (ICAM-1) and vascular cell adhesion molecule 1 (VCAM-1) [24,25,26].

Periodontitis can aggravate endothelial dysfunction through systemic inflammation, LPS exposure, oxidative stress, TMAO signaling, immune-cell activation and direct interactions between periodontal pathogens and endothelial cells. Gram-negative periodontal organisms may adhere to or invade endothelial cells, induce platelet aggregation and facilitate thrombus formation [30]. These mechanisms position endothelial dysfunction as the vascular interface through which oral–gut dysbiosis may translate into premature atherosclerotic disease.

## 6. Vascular Remodeling and Premature Coronary Artery Disease

### 6.1. Plaque Initiation and Progression

Atherosclerosis begins with endothelial dysfunction and increased endothelial permeability, which facilitate retention and modification of low-density lipoprotein within the arterial intima [31]. Oxidized and otherwise modified LDL particles promote endothelial activation, monocyte recruitment and macrophage lipid uptake. Lipid-laden macrophages become foam cells and form fatty streaks, the earliest visible atherosclerotic lesions [31]. Continued lipid accumulation and inflammatory activation lead to plaque enlargement, necrotic core formation, fibrous cap remodeling and vulnerability to rupture [31,32].

Periodontal dysbiosis may influence several steps in this cascade. *P. gingivalis* has been detected within atherosclerotic plaques in humans and animal models, supporting the possibility of direct microbial participation in vascular lesions [28]. Periodontal pathogens, including *P. gingivalis*, *Tannerella forsythia* and *Prevotella intermedia*, have also been identified in periodontal pockets and vascular tissue from patients with atherosclerosis or abdominal aortic aneurysm [33]. While detection does not prove causality, it strengthens the biological plausibility of a direct oral–vascular microbial connection.

### 6.2. Imaging Evidence of Subclinical Vascular Disease

Subclinical vascular imaging provides important evidence linking periodontal disease with early atherosclerosis. Carotid intima-media thickness (CIMT) is the most widely studied surrogate marker, and higher CIMT has been reported in association with more severe periodontal disease [6]. Measures of cumulative periodontal destruction, including attachment loss combined with tooth loss, have been independently associated with subclinical large-artery atherosclerosis, suggesting that long-term periodontal burden may be more relevant than current inflammatory activity alone [34].

Increased periodontal pocket depth has been associated with higher CIMT, elevated hsCRP and increased monocyte and neutrophil counts, supporting a link between periodontal inflammation, systemic immune activation and vascular changes [35]. Arterial stiffness has also been investigated using pulse wave velocity (PWV), with higher PWV reported in patients with periodontitis, including cohorts with systemic inflammatory disease [36]. Available studies examining periodontitis in relation to coronary artery calcium (CAC) or coronary computed tomography angiography (CCTA)-derived plaque characteristics remain limited and heterogeneous. Some investigations have not demonstrated an independent association after adjustment for conventional risk factors, whereas selected cohorts with established coronary disease have shown relationships between periodontal inflammatory burden and high-risk plaque features. These discrepant findings highlight the need for prospective studies using standardized periodontal definitions, contemporaneous imaging, and rigorous confounder adjustment, particularly in younger populations.

### 6.3. Evidence Specific to Premature CAD: Direct Data, Extrapolation, and Uncertainty

Premature CAD has clinical and epidemiological features that distinguish it from later-onset disease. Young patients often have high rates of smoking, dyslipidemia, obesity, family history and hypertension, whereas diabetes may be less frequent than in older cohorts [37,38]. Illicit drug use and psychosocial stressors may also contribute in selected populations [37]. Clinical presentation is often dominated by myocardial infarction rather than stable angina, and a previous history of angina may be absent [37].

Young patients with coronary events are not protected from adverse outcomes. Mortality may be comparable to that observed in older groups in some settings, and premature CAD is associated with an increased risk of recurrent myocardial infarction, stroke and repeat revascularization [38,39]. These findings highlight the need for earlier identification of non-traditional and modifiable contributors to vascular risk. The oral–gut–vascular axis may be particularly relevant in PCAD because chronic microbial and inflammatory stimuli can accelerate vascular injury before decades of conventional cardiometabolic exposure have accumulated.

Importantly, direct human evidence linking periodontal dysbiosis to premature coronary artery disease through a gut-mediated pathway remains limited. Most available data derive from experimental models, cross-sectional human studies, or studies of atherosclerosis conducted in unselected adult populations. Therefore, the proposed relevance of the oral–gut–vascular axis to PCAD is based on the convergence of three complementary evidence streams: longitudinal associations between early-life oral infection and later subclinical atherosclerosis; observational associations between periodontitis and vascular disease in the general adult population; and mechanistic studies demonstrating oral–gut microbial translocation, barrier dysfunction, endotoxemia, and vascular inflammation. This distinction is essential because biological plausibility does not by itself establish causality or PCAD-specific clinical relevance.

Accordingly, the oral–gut–vascular axis should currently be regarded as a testable integrative model, not an established clinical pathway in PCAD. The evidence is strongest for a general periodontitis–ASCVD association, weaker for gut-mediated causal pathways, and weakest for direct prediction or treatment of premature coronary events.

## 7. Emerging Biomarkers of the Oral–Gut–Vascular Axis

A precision medicine approach requires measurable biomarkers that connect periodontal status, gut barrier integrity, microbial metabolism, systemic inflammation and vascular injury. At present, no single biomarker can define the oral–gut–vascular axis. A multimodal panel is more plausible, combining oral, fecal, plasma and imaging markers.

Oral biomarkers may include periodontal clinical indices, salivary inflammatory mediators, pathogen-specific microbial signatures and measures of periodontal destruction. Gut-related markers may include fecal microbial diversity, enrichment of oral taxa in stool, markers of intestinal permeability and microbial metabolite profiles. Circulating markers may include hsCRP, IL-6, TNF-α, LPS, LBP, soluble CD14, TMAO, oxidized LDL, ICAM-1, VCAM-1 and P-selectin. Vascular assessment may include flow-mediated dilation, PWV, CIMT, CAC and CCTA. The integration of these markers could identify young individuals with disproportionate inflammatory and microbiome-related vascular risk.

Among these candidates, hsCRP and IL-6 are clinically familiar markers of systemic inflammation, while TMAO offers a mechanistic link between microbial metabolism and atherothrombotic pathways. LPS and LBP may reflect endotoxemia, although technical variability and standardization issues remain. Zonulin and other permeability-related markers require cautious interpretation but may help characterize barrier dysfunction when combined with microbiome and inflammatory data. Salivary diagnostics are attractive because they are non-invasive and can be integrated into dental screening workflows.

Critical interpretation is essential because most candidate biomarkers are not specific to the proposed axis. hsCRP and IL-6 reflect inflammation from multiple sources; TMAO is strongly influenced by renal function, diet, age, and hepatic metabolism; and LPS, LBP, and permeability assays show substantial pre-analytical and analytical variability. Clinical translation will therefore require evidence of incremental discrimination, calibration, and decision benefit beyond established cardiovascular risk factors, rather than association alone.

## 8. Therapeutic Perspectives: From Periodontal Treatment to Precision Microbiome Modulation

### 8.1. Periodontal Therapy as an Upstream Intervention

The most direct therapeutic implication of the oral–gut–vascular axis is that periodontal disease represents a treatable source of chronic microbial and inflammatory exposure. Periodontal treatment can reduce local inflammation and may decrease systemic inflammatory burden [12,40]. Interventions such as scaling and root planing, plaque control, adjunctive antimicrobial approaches and long-term periodontal maintenance may therefore influence systemic pathways relevant to vascular health.

Clinical studies have reported reductions in inflammatory and thrombotic biomarkers after periodontal therapy, including CRP, fibrinogen, cytokines, white blood cell counts and systolic blood pressure, together with improvements in surrogate measures of endothelial dysfunction [40]. Nevertheless, current evidence remains insufficient to conclude that periodontal treatment alone prevents hard cardiovascular outcomes. This limitation should not minimize the clinical relevance of periodontal care, but it underscores the need for adequately powered randomized trials using standardized periodontal, microbiome, biomarker and vascular endpoints.

Preventive oral healthcare may also be relevant. Regular dental attendance has been associated with lower prevalence of atherosclerosis [41], and large population-based data suggest that dental scaling is associated with reduced risk of peripheral arterial occlusive disease [42]. These associations may reflect improved periodontal health, healthier behaviors or residual confounding; however, they support the broader concept that oral health should be considered within cardiovascular prevention frameworks.

### 8.2. Microbiome Modulation and Barrier Restoration

The therapeutic implications of the oral–gut–vascular axis extend beyond periodontal therapy to emerging strategies aimed at modulating microbial function and restoring intestinal barrier integrity. Potential strategies include dietary interventions that reduce pro-atherogenic microbial metabolite production, increased intake of fermentable fibers to support short-chain fatty acid generation, probiotics or next-generation probiotics targeting barrier integrity, prebiotics, synbiotics, postbiotics and interventions designed to restore mucosal immune tolerance. These approaches remain investigational in the specific context of periodontitis-associated PCAD, but they align with the mechanistic pathways summarized above.

TMAO-targeted approaches are of particular interest. Precision nutrition may reduce exposure to substrates that promote microbial TMA production in selected high-risk individuals, while future strategies may target microbial enzymes involved in TMA generation or host pathways regulating FMO3. Barrier-focused interventions could aim to strengthen tight junctions, reduce endotoxemia and lower systemic inflammatory tone. Importantly, microbiome modulation should not be presented as a replacement for established cardiovascular prevention, but as a potential adjunct to lipid control, blood pressure management, smoking cessation, weight optimization and periodontal care.

### 8.3. Integrated Cardio–Dental Screening

The oral–gut–vascular axis supports a model of integrated cardio-dental screening. In young adults with PCAD, strong family history, unexplained residual inflammatory risk or subclinical atherosclerosis, periodontal assessment may identify a modifiable inflammatory burden. Conversely, patients with severe periodontitis, early tooth loss or high periodontal inflammatory load may benefit from cardiovascular risk assessment, especially when additional risk factors are present.

A practical screening pathway could include periodontal examination, salivary or systemic inflammatory markers, conventional cardiovascular risk assessment and selective vascular imaging in higher-risk individuals. In research settings, fecal microbiome profiling, metabolomics and endothelial function testing could clarify mechanisms and identify treatable phenotypes. This interdisciplinary approach is clinically attractive because it combines non-invasive assessment, modifiable exposures and prevention-oriented care.

At present, these concepts should not be interpreted as support for routine microbiome sequencing, TMAO testing, or vascular imaging solely because periodontitis is present. Cardio–dental collaboration is justified for recognition and treatment of established oral and cardiovascular risk factors, whereas microbiome-guided screening remains a research strategy until prospective clinical utility is demonstrated.

Table 1 separates the representative evidence signal from the main limitation that constrains causal interpretation for PCAD.

These markers should be regarded as candidate mechanistic or translational readouts; none currently defines or validates the oral–gut–vascular axis in clinical practice.

## 9. Artificial Intelligence, Multi-Omics and Precision Risk Prediction

Artificial intelligence (AI) and machine-learning methods may facilitate the translation of the oral–gut–vascular axis by integrating periodontal indices, dental imaging, salivary and fecal metagenomic profiles, plasma metabolomics, inflammatory and endotoxemia-related biomarkers, vascular imaging, and conventional cardiovascular risk factors into multimodal prediction models [43,44].

Recent cross-cohort studies indicate that oral metagenomic signatures can discriminate periodontitis from periodontal health, while integration of oral microbiome features with clinical variables may improve atherosclerotic cardiovascular disease risk classification; however, these findings remain proof-of-concept and have not been externally validated in patients with premature coronary artery disease [45,46]. Such approaches may be particularly relevant in younger adults, in whom age-weighted cardiovascular algorithms may underestimate early atherosclerotic burden, although the incremental predictive value of microbiome-derived variables must be demonstrated beyond established risk scores. Robust model development requires appropriate handling of sparse, compositional, and batch-sensitive microbiome data, strict prevention of data leakage, management of class imbalance, and assessment of both discrimination and calibration [47,48,49,50].

Explainable AI frameworks should identify biologically plausible microbial pathways, metabolites, and host interactions rather than generate opaque probability estimates, and any derived signatures should be independently validated using functional, longitudinal, or experimental data [51]. Before clinical implementation, candidate models require external multicenter validation, assessment of fairness across demographic and clinical subgroups, and prospective evaluation of their effect on cardio-dental decision-making and patient outcomes [49,50,52].

A potentially useful strategy for dimensionality reduction is the use of microbiome dysbiosis indices rather than individual taxa. The Subgingival Microbial Dysbiosis Index (SMDI), derived from machine-learning identification of health- and periodontitis-associated taxa, provides a continuous summary measure of periodontal microbial imbalance. Importantly, subsequent analyses suggest that related dysbiosis signatures can also be detected in saliva, supporting its potential use as a more accessible oral sampling compartment. Analogous indices have been proposed for intestinal dysbiosis, although methodological heterogeneity has prevented the emergence of a universally accepted gut dysbiosis score.

For the oral–gut–vascular axis, cross-niche or composite dysbiosis scores are conceptually attractive because they could quantify concordant ecological disruption across oral and intestinal compartments. However, such indices should not be assumed to be interchangeable across niches or populations and require external validation, harmonized sequencing and preprocessing, and demonstration of incremental predictive value beyond conventional cardiovascular and periodontal variables [53].

## 10. Knowledge Gaps and Future Research Priorities

Interpretation of oral–gut microbiome studies requires substantial methodological caution. Detection of oral-associated taxa in fecal samples does not necessarily demonstrate stable intestinal engraftment and may reflect transient passage, ecological overlap, or methodological limitations. Strain-resolved shotgun metagenomics, longitudinal paired oral–stool sampling, microbial source tracking, and functional metagenomic or metabolomic analyses are required to distinguish true colonization from transient detection. Moreover, associations may be influenced by diet, smoking, diabetes, obesity, socioeconomic status, oral hygiene, medication exposure, renal function, and healthcare utilization. These factors should be considered explicitly in future prospective and interventional studies.

Several knowledge gaps limit the current translation of the oral–gut–vascular axis into clinical practice. First, much of the mechanistic evidence comes from animal models or observational human studies. Prospective longitudinal cohorts are needed to determine whether periodontal dysbiosis predicts incident PCAD independently of conventional risk factors and whether gut dysbiosis or microbial metabolites mediate this association.

Second, causality remains incompletely established. Future studies should use integrated designs combining periodontal phenotyping, oral and gut microbiome sequencing, metabolomics, permeability markers, inflammatory profiling and vascular imaging. Mendelian randomization, causal mediation analysis and interventional trials may help distinguish correlation from causal contribution. Third, periodontal treatment trials should include vascular endpoints beyond inflammatory biomarkers, such as PWV, CIMT, CAC progression or CCTA-derived plaque features.

Fourth, biomarker standardization is required. Measurements of LPS, zonulin, TMAO and salivary microbial signatures vary across platforms and populations. Reproducible thresholds and clinically meaningful changes must be defined. Fifth, the specific relevance of this axis in young adults is underexplored. PCAD-focused studies are required because mechanisms driving early atherosclerosis may differ from those in older populations with decades of metabolic and hemodynamic exposure.

Finally, therapeutic research should evaluate combined strategies. Periodontal therapy, dietary modulation, smoking cessation, conventional cardiovascular prevention and microbiome-targeted interventions may have additive or synergistic effects. Precision approaches should identify which patients are most likely to benefit from each intervention and which biomarkers indicate treatment response.

## 11. Conclusions

Periodontal dysbiosis may contribute to premature atherosclerosis through a biologically plausible, but not yet causally or clinically validated, oral–gut–vascular axis. This axis integrates microbial translocation, oralization of the gut microbiome, intestinal barrier dysfunction, endotoxemia, TLR-mediated inflammation, TMAO metabolism, oxidative stress, immune dysregulation and endothelial dysfunction. These mechanisms converge on plaque initiation, progression and instability, providing a potential explanation for part of the residual inflammatory risk observed in young adults with PCAD.

The current evidence supports periodontal disease as a modifiable inflammatory condition with systemic vascular relevance, but does not yet establish gut mediation, PCAD-specific risk prediction, or reduction in hard cardiovascular outcomes through periodontal or microbiome-targeted interventions. Future research should prioritize prospective PCAD cohorts, mechanistic human studies, standardized biomarker panels, vascular imaging endpoints and AI-assisted integration of oral, gut, metabolic and cardiovascular data. In clinical practice, closer collaboration between cardiology, dentistry, microbiology and preventive medicine may improve early identification of individuals with microbiome-related vascular risk and support more comprehensive prevention strategies.

## Figures and Tables

**Table 1:** Evidence map of the proposed oral–gut–vascular axis: representative signals, critical limitations, and relevance to premature coronary artery disease.

Evidence Domain (Evidence Base)	Candidate Biomarkers/Readouts	Representative Data/Signal	Critical Interpretation for PCAD
Direct oral-vascular dissemination (human observational/mechanistic) [6,13,27,33]	Periodontal indices, salivary microbial load/signatures, circulating microbial DNA/products	Transient bacteremia, circulating microbial products, systemic CRP/cytokine elevation, and periodontal taxa or DNA detected in vascular tissue.	Supports biological plausibility, but detection does not prove organism viability, vascular invasion, or causal contribution. Prospective PCAD-specific data are lacking.
Oral–gut translocation and barrier dysfunction (experimental + emerging human) [7,18,19,20,21]	Paired oral–stool strain tracking, fecal oral taxa, LPS, LBP, sCD14, permeability markers	Oral taxa are detectable in stool; experimental periodontal exposure alters gut diversity, intestinal permeability, and circulating LPS.	Human strain-resolved source tracking is sparse, and stool detection may reflect transient passage. Gut mediation of PCAD remains unproven.
Endotoxemia and TLR2/TLR4 signaling (mechanistic) [21,22]	LPS, LBP, sCD14, hsCRP, IL-6, TNF-α	LPS-LBP-CD14 signaling activates TLR2/TLR4, NF-kB/MAPK, IL-1β, IL-6, TNF-α, monocytes, and endothelial cells.	Mechanistically coherent but nonspecific. Endotoxemia is influenced by obesity, diabetes, diet, and other barrier disorders; causal mediation has not been shown.
TMA/TMAO metabolism (experimental) [20,23]	Plasma TMAO, metabolomic signatures; experimental FMO3 readouts	Experimental periodontitis increases TMAO/FMO3 and worsens plaque; TMAO promotes endothelial activation, foam-cell formation, and platelet reactivity.	A candidate mediator, not an oral-specific biomarker. Levels are strongly influenced by diet, renal function, age, hepatic metabolism, and the wider gut microbiome.
Endothelial and vascular phenotype (human observational/surrogate) [24,25,26,27,34,35,36]	Oxidized LDL, NO-related measures, ICAM-1, VCAM-1, Flow-mediated dilation (FMD) CIMT, PWV, CAC, CCTA	Periodontitis is linked to ROS and NO imbalance, ICAM-1/VCAM-1 activation, higher CIMT and PWV, and selected high-risk plaque associations.	Evidence relies mainly on surrogate endpoints and heterogeneous confounder adjustment; event reduction and specific relevance to young adults are unproven.
Periodontal intervention (human interventional) [12,40,41,42]	CRP, fibrinogen, cytokines, FMD/PWV	Periodontal therapy may reduce CRP, fibrinogen, cytokines, and white-cell counts and improve selected endothelial or vascular surrogate measures.	Trials are heterogeneous and underpowered for MACE. Biomarker improvement cannot be interpreted as proven prevention of PCAD or cardiovascular events.

**Abbreviations**: CD14, cluster of differentiation 14; CIMT, carotid intima–media thickness; CRP, C-reactive protein; DNA, deoxyribonucleic acid; FMO3, flavin-containing monooxygenase 3; ICAM-1, intercellular adhesion molecule 1; IL, interleukin; LBP, lipopolysaccharide-binding protein; LPS, lipopolysaccharide; MACE, major adverse cardiovascular events; MAPK, mitogen-activated protein kinase; NF-κB, nuclear factor kappa B; NO, nitric oxide; PCAD, premature coronary artery disease; PWV, pulse wave velocity; ROS, reactive oxygen species; TLR, Toll-like receptor; TMA, trimethylamine; TMAO, trimethylamine N-oxide; TNF-α, tumor necrosis factor alpha; VCAM-1, vascular cell adhesion molecule 1.

## Data Availability

No new data were created or analyzed in this study. Data sharing is not applicable to this article.

## Abbreviations

The following abbreviations are used in this manuscript:

AI	Artificial Intelligence
ASCVD	Atherosclerotic Cardiovascular Disease
CAC	Coronary Artery Calcium
CAD	Coronary Artery Disease
CD14	Cluster of Differentiation 14
CIMT	Carotid Intima–Media Thickness
CCTA	Coronary Computed Tomography Angiography
CRP	C-Reactive Protein
eNOS	Endothelial Nitric Oxide Synthase
FMD	Flow-mediated dilation
FMO3	Flavin-Containing Monooxygenase 3
HGF	Hepatocyte Growth Factor
hsCRP	High-Sensitivity C-Reactive Protein
ICAM-1	Intercellular Adhesion Molecule-1
IL-1	Interleukin-1
IL-1β	Interleukin-1 Beta
IL-6	Interleukin-6
IL-8	Interleukin-8
LBP	Lipopolysaccharide-Binding Protein
LDL	Low-Density Lipoprotein
LPS	Lipopolysaccharide
MAPK	Mitogen-Activated Protein Kinase
MD-2	Myeloid Differentiation Protein 2
NF-κB	Nuclear Factor Kappa B
NO	Nitric Oxide
OMV	Outer Membrane Vesicle
PCAD	Premature Coronary Artery Disease
PWV	Pulse Wave Velocity
ROS	Reactive Oxygen Species
SMDI	The Subgingival Microbial Dysbiosis Index
Th17	T Helper 17 Cell
TLR	Toll-Like Receptor
TLR2	Toll-Like Receptor 2
TLR4	Toll-Like Receptor 4
TMA	Trimethylamine
TMAO	Trimethylamine N-oxide
TNF-α	Tumor Necrosis Factor Alpha
Treg	Regulatory T Cell
VCAM-1	Vascular Cell Adhesion Molecule-1
